# Development of Laser Processing Carbon-Fiber-Reinforced Plastic

**DOI:** 10.3390/s23073659

**Published:** 2023-03-31

**Authors:** Zhonghe Wang, Yao Ma, Boshi Yuan, Chunting Wu, Changqing Li, Shuwei Sun

**Affiliations:** 1 Jilin Key Laboratory of Solid-State Laser Technology and Application, Changchun University of Science and Technology, Changchun 130022, China; w1125740757@126.com (Z.W.); mayao@cust.edu.cn (Y.M.); yuanboshi1986@163.com (B.Y.); 2 Scientific and Technological Innovation Center, Beijing 100012, China

**Keywords:** carbon-fiber-reinforced plastic, laser processing, high efficiency, processing technology, thermal damage, high precision

## Abstract

Due to its exceptional advantages, such as high specific strength, high specific modulus, and good fatigue resistance, carbon-fiber-reinforced plastic (CFRP) is frequently utilized in aerospace, aviation, automotive, rail transportation, and other areas. Composite components typically need to be joined and integrated. In the equipment manufacturing industry, the most used methods for processing composite components are cutting, drilling, and surface treatment. The quality of CFRP is significantly impacted by traditional mechanical processing, causing flaws like delamination, burrs, and tears. Laser processing technology has emerged as a crucial method for processing CFRP for its high quality, non-contact, simple control, and automation features. The most recent research on the laser processing of CFRP is presented in this paper, supporting scientists and engineers who work in the field in using this unconventional manufacturing technique. This paper gives a general overview of the key features of laser processing technology and the numerous machining techniques available. The concepts and benefits of laser processing technology are discussed in terms of the material properties, mode of operation, and laser characteristics, as well as the methods to achieve high efficiency, low damage, and high precision. This paper reviews the research development of laser processing of carbon-fiber-reinforced plastics, and a summary of the factors affecting the quality of CFRP laser processing. Therefore, the research content of this article can be used as a theoretical basis for reducing thermal damage and improving the processing quality of laser-processed composite materials, while, on this basis, we analyze the development trend of CFRP laser processing technology.

## 1. Introduction

Carbon-fiber-reinforced plastic (CFRP) is one of the most advanced composite materials available nowadays. CFRP is molded by epoxy as the matrix and carbon fiber as the reinforcement. Its low density (roughly 1.3–2.0 g/cm^3^, only about one-quarter of ordinary steel), high specific strength, designability, corrosion resistance, and other advantages are unmatched by other fiber composite materials. CFRP is widely used as lightweight materials in the aerospace, automotive, wind-power, construction, and other industrial fields [1,2,3,4,5,6,7].

CFRP is often prepared entirely at once by using hot press cans, film pressure, etc. [8]. However, further processing of CFRP, such as trimming, drilling, cutting, and joining, is necessary because of the assembly requirements of manufacturing [9]. The two types of secondary processing methods are conventional mechanical processing and special processing. Drilling, milling, sawing, and wire cutting are methods of traditional mechanical processing technologies that can harm materials by causing fractures, rips, and delamination [10,11]. Therefore, researchers developed novel processing technologies, such as laser processing, abrasive water-jet processing [12], electrical discharge machining (EDM) processing [13], ultrasonic vibration processing [14], novel processing technologies, etc. However, due to CFRP’s high strength, high hardness, high wear resistance, multi-layer diverse materials, and other variables, as well as the properties of inhomogeneity and anisotropy, it becomes a challenging material to process. Despite reducing mechanical damage, abrasive water-jet processing still causes processing defects such as kerf deformation and material delamination, and it has certain limitations for cutting complex parts. The EDM process, on the other hand, produces little mechanical damage but has low machining efficiency and poor machining accuracy. Although the ultrasonic vibration processing method reduces cutting force, fiber delamination pull-out and internal cracking still occur [12,13,14].

As a mature processing method, laser processing has no tool loss, fast processing speed, and easy automation control in the process. Many scholars have compared laser processing methods and other special processing methods. In terms of processing damage and defects, laser processing has the least impact on the fatigue strength and tensile strength of fiber composites. In terms of economic efficiency and cost, laser processing is the most efficient and cost-effective method of processing [15,16,17,18]. In terms of processing, laser processing is a highly controllable contactless processing that can be used in drilling, polishing, cutting, milling, surface pre-treatment, and almost all common types of work. Laser processing can be combined with other energy fields, such as water jets, ultrasound, electromagnetic fields, and other auxiliary laser processing compound energy-field processing. Many countries have realized the industrial production of laser processing CFRP with easy automation control and high flexibility. A five-axis laser cutting machine for CFRP of 3D molded samples is created in Japan [19], laser robots are used in Germany to cut high-end automotive carbon fiber composite parts [20], and the near-infrared nanosecond laser robot with a power of 1.5 kW has completed the cutting process of a space-curved vehicle structure made of CFRP material with a maximum thickness of 5 mm [21]. Researchers in Russia used laser processing to create the lightest aerospace solar panel [22].

With the tremendous advancement of laser technology, there are increasingly more types of lasers, and dozens of different types of lasers are used in CFRP processing. According to the classification of excitation material, they can be divided into semiconductor diode laser, solid-state laser, and gaseous laser. They are classified into ultraviolet laser, visible laser, infrared laser, and so on based on wavelength. Continuous laser, long pulse laser (microsecond, millisecond), short pulse laser (nanosecond), and ultrafast laser are the output modes (picosecond and femtosecond).

**Figure 1:**
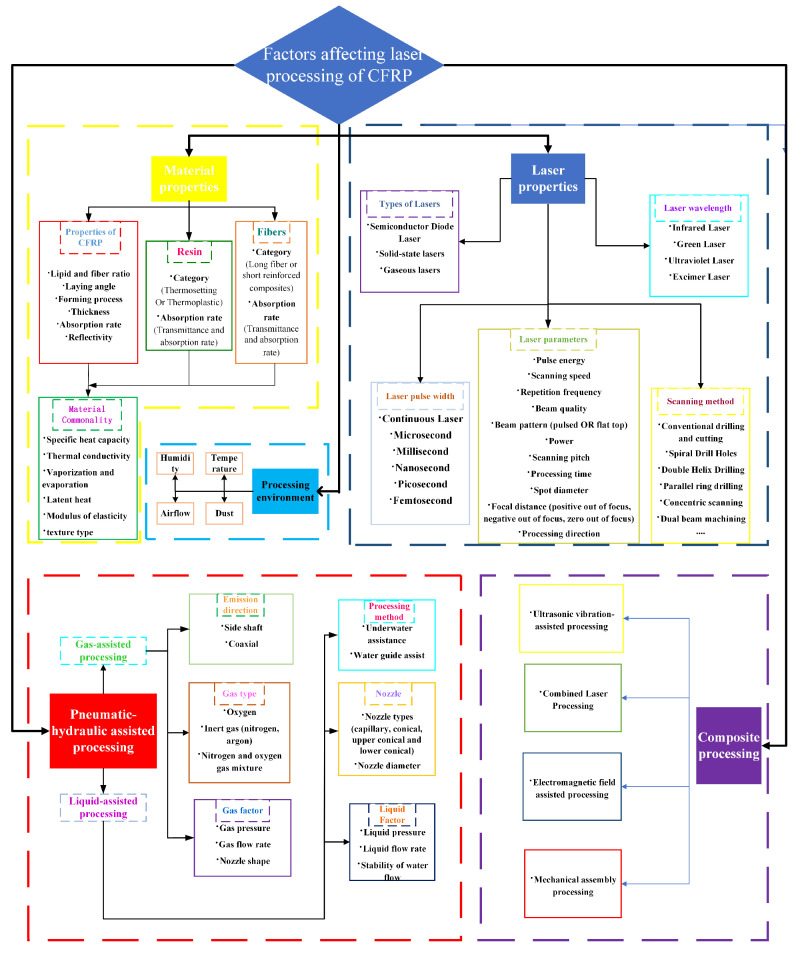
Influencing factors of CFRP laser processing.

The first ruby laser in the world was created in 1960, and laser cutting was first used to drill holes in diamond molds in 1965. Since then, laser irradiation processing has been used to process materials in a variety of ways in industry. Tagliaferri et al. [23] investigated the laser processing of CFRP for the first time in 1985. In the past few decades, there have been both challenges and opportunities for the laser processing of composite materials. First, resin materials are classified as thermosetting and thermoplastic materials. Fiber-reinforced composites can be divided into short-fiber-reinforced composites with discontinuous short fibers as the reinforcing phase and long-fiber-reinforced composites with continuous long fibers as the reinforcing phase. Second, the physical properties of carbon fiber and epoxy resin differ greatly. For example, the gasification temperature of carbon fiber exceeds 3300 °C, whereas the resin begins to gasify at 500 °C. On the other hand, the thermal conductivity of the resin is only 0.2 W/(m·K), while the carbon fiber is anisotropic. The axial thermal conductivity (50 W/(m·K)) is much greater than the radial (5 W/(m·K)) [24]. Due to the variability of composite materials, laser processing can lead to severe thermal damage such as a heat-affected zone (HAZ), fiber pull-out, laminar delamination, and fiber-end expansion [25]. These damages not only affect the appearance and quality of the structure being machined, but also seriously affect the geometric accuracy and quality of the structure being machined, despite having a lower impact on the mechanical properties than the rest of the special and conventional machining methods. According to studies, the main reason for the decline in mechanical properties of CFRP is that HAZ makes the exposed carbon fibers unable to withstand the load. The bending strength, compressive strength, and tensile strength of CFRP all decrease linearly with the expansion of a HAZ region [17]. Because of the significant influence of the heat-affected zone, the size of the HAZ has now become a criterion for determining the processing quality of CFRP. According to the statistics of research in the field of CFRP laser processing carried out by EL-Hofy [26], the research on the HAZ accounts for 76.8%. Although it is nearly impossible to get undamaged CFRP samples in laser processing, the quality of the samples can be improved by optimizing the process parameters (laser pulse width, wavelength, scanning speed, etc.) [27]. Carbon fiber can endure relatively high temperatures, while the resin matrix will experience mechanical degradation as temperature increases. The resin is prone to water absorption and deliquescence, which causes the expansion of the HAZ during processing and lowers the processing quality [28,29,30,31]. Shorter wavelengths and narrower pulse widths can lessen the thermal impact and thermal diffusion damage. The gas- and liquid-assisted laser processing can be used to cool the processing area and reduce heat accumulation. The width of the HAZ during the laser processing of CFRP can be influenced by a variety of factors. Figure 1 provides an overview of the factors influencing laser processing.

**Figure 2:**
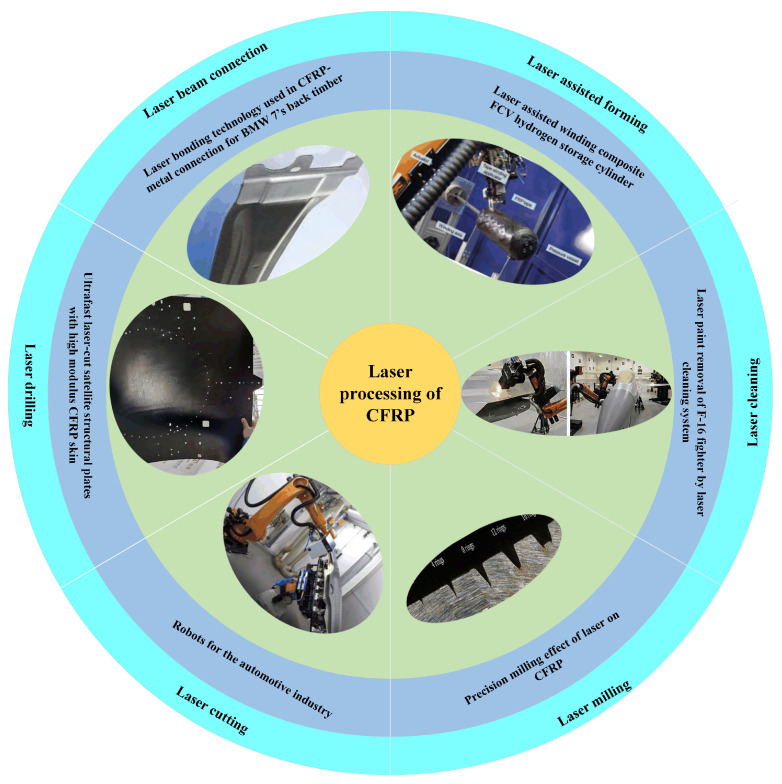
Laser technology used in CFRP [5,20,32,33,34].

However, for continuous lasers and millisecond or even nanosecond short-pulse lasers processing, the thermal damage region is large. As a new processing technology, ultrashort pulse laser processing with extreme physical characteristics, such as picosecond laser and femtosecond laser, has the advantages of tiny scale, strong controllability, environmental friendliness, no restriction of material types, non-contact processing, and no heat transfer. Currently, laser processing has emerged as a viable method for overcoming the technical barrier of the precision processing of fiber-reinforced composites. This technology has been successfully applied to surface cleaning and activation, surface metallization, and other manufacturing techniques, meeting new requirements like connection or functional surface preparation. It is anticipated that laser processing can overcome the shortcomings in processing accuracy and damage. Nowadays, laser processing on CFRP has a wide range of applications, such as drilling, cutting, milling [32], cleaning, connecting [33], auxiliary molding [34], and other processes [35], as indicated in Figure 2.

This article summarizes the main factors affecting the processing quality of laser cutting CFRP. First, the process of laser and CFRP processing and the mechanism of action are discussed. Then the laser wavelength, pulse width, and other laser characteristics, as well as the scanning speed, repetition frequency, and other laser parameters on the processing quality and processing efficiency of the law, was analyzed. New laser processing methods that can improve processing quality are summarized. To further elucidate the CFRP laser removal mechanism, the progress of numerical simulation studies of CFRP laser processing of CFRP is also reviewed. Finally, the development trend of laser processing of CFRP is foreseen. This paper can provide a reference for the development of laser processing CFRP technology.

## 2. CFRP and Laser Interaction Mechanism

### 2.1. The Basic Process of Laser Processing CFRP

**Figure 3:**
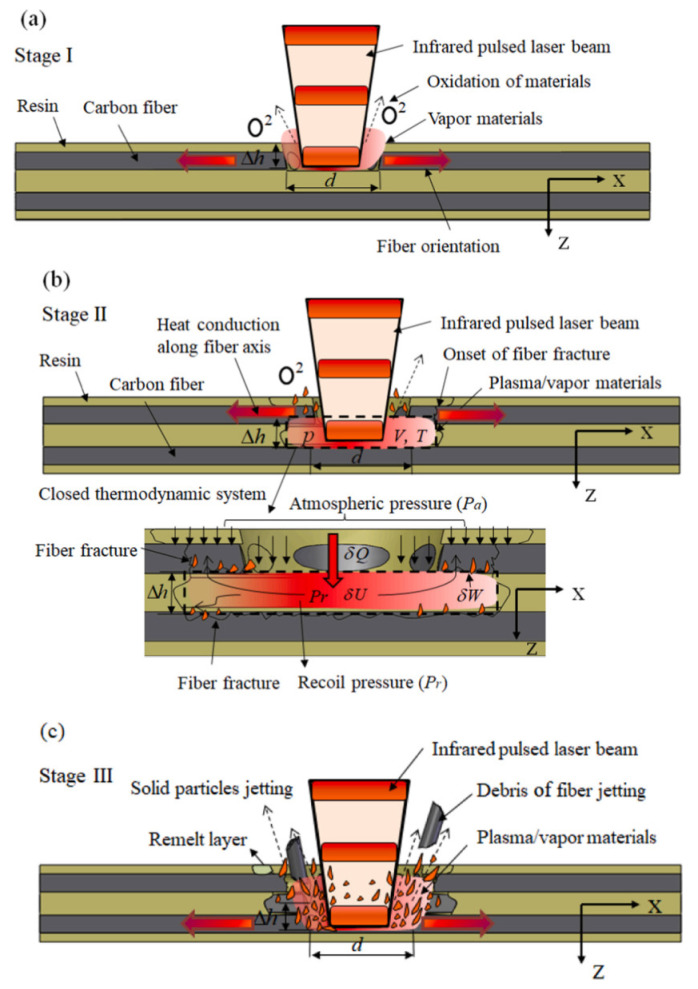
Schematic diagram of material removal for different stages in pulsed-laser machining of CFRP composites: (**a**) stage I; (**b**) stage II; (**c**) stage III [36].

The surface of CFRP is exposed to high-energy density laser irradiation during processing. Thermal deterioration and evaporation, which can be further broken down into heating and sublimation phases, occur initially on the substrate. Carbon fibers are oxidized and break down in the presence of high temperatures and oxygen to produce organic gases like CO, carbon dioxide, etc., forming mixed vapor. When the vapor on the surface continues to absorb heat and the temperature rises above the sublimation point, it will form the plasma. Following this, the volume of vapor/plasma rapidly expands, and the rebound pressure created by the vapor continues to act on the outside. Subsequently, the fibers on both sides break and start splashing out in large quantities. This state is maintained until the end of the laser pulse. The melted liquid-phase resin will clump on the substrate surface, forming a recast layer. Therefore, in the process of laser processing CFRP, there are three forms of interaction between the laser beam and CFRP: ablation, combustion, and mechanical effect caused by recoil pressure. According to the processing duration and energy density, the laser processing process can be divided into the following stages: evaporation and oxidation stage (stage I), gas expansion stage (stage II), and mechanical removal stage (stage III), as illustrated in Figure 3. As can be observed from the processing process, laser parameters are the main factors affecting the quality of CFRP processing [36,37].

### 2.2. Materials Evolution in Laser Processing

The energy transfer between the laser and the composite is affected by the laser wavelength and pulse width. The process contains a variety of ablative damage behaviors, and the processing mechanism is rather complicated.

**Figure 4:**
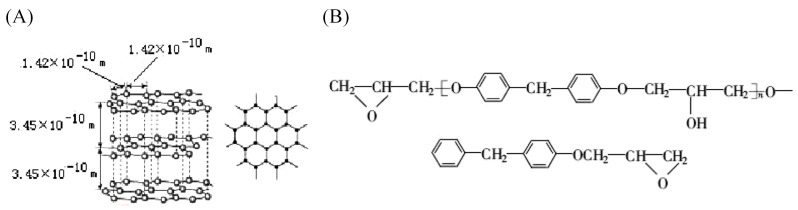
Microstructure of carbon fiber and epoxy resin: (**A**) microstructure of carbon fiber; (**B**) main structure of epoxy resin [38].

The microstructure of carbon fiber is a layered structure, as shown in Figure 4A. Each carbon atom is connected to the other three carbon atoms by two C–C bonds and one C=C bond. In its natural state, the bond energy of the C–C bond is 3.45 eV, whereas of the C=C bond is 6.34 eV. Figure 4B illustrates the basic structure of epoxy resin. It primarily consists of C–O, C–H, C–C, and C=C bonds, with a bond energy of 7.56 eV for the C–O bond and 4.30 eV for the C–H bond [38].

**Figure 5:**
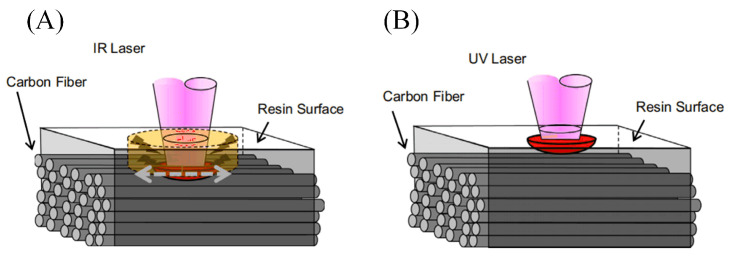
Laser cutting process of CFRP, which depends on the wavelength of the laser: (**A**) infrared laser and (**B**) UV laser processing CFRP mechanism [40].

The processing mechanism of CFRP by an IR laser and a UV laser is different. Compared with the ultraviolet laser, resin has greater transmittance to an IR laser, and the single photon energy of the IR laser with a wavelength of 1064 nm is 1.17 eV, which is insufficient to break the chemical bond. Therefore, the IR laser can remove the carbon fiber by gasification through a photothermal mechanism, and the resin is eliminated by the combined action of the heat conduction of the carbon fiber and the gas pressure. When the wavelength of the UV laser is 355 nm, the single photon energy is 3.49 eV, which is higher than the C–C bond energy of 3.45 eV found in the main structure of both materials. Therefore, UV laser processing is a photochemical reaction process that can break the C–C bond of carbon fiber and epoxy resin directly, achieving the “cold processing” of the material. The processing principle of both is shown in Figure 5 [38,39,40,41].

**Figure 6:**
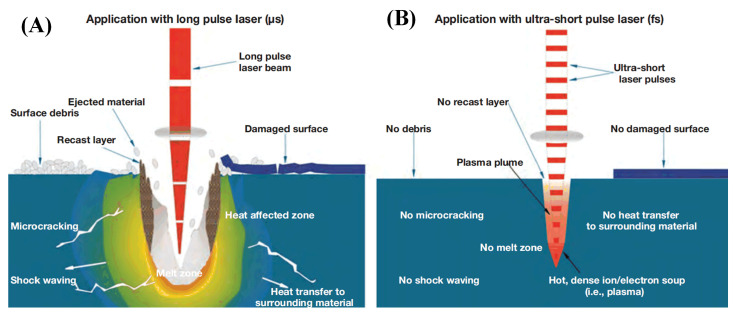
Schematic processing comparison of microsecond to femtosecond lasers: (**A**) long-pulse laser and (**B**) femtosecond laser processing [42].

According to the pulse width of the laser, the laser processing process can be divided into thermodynamic and non-thermodynamic processes. The first category includes continuous lasers, long-pulse lasers, and short-pulse lasers. When the pulse width is greater than 10^−11^ s, several heat transfer and diffusion processes will occur when the material absorbs the laser energy, including electron excitation, electron–phonon relaxation, and phonon–phonon relaxation. Therefore, in the process of laser processing, the melting, evaporation, and removal of the material are achieved by thermal effects, which cause serious thermal effects and harm to the edge of the laser-burned area, as shown in Figure 6A. When the CFRP is processed by the picosecond and femtosecond lasers, the laser pulse duration is shorter than the electron–lattice energy relaxation period. The laser energy has been absorbed by the electrons before the energy coupling between electron and lattice, and the lattice and electrons are in a non-equilibrium state. The “cold processing” mechanism of the interaction between the ultrashort-pulse laser and material is shown in Figure 6B. During the pulse duration, the lattice motion and heat conduction are negligible. The material undergoes an instantaneous non-thermal phase change, and the material is ejected with the formed plasma before the laser energy can diffuse to the periphery. This prevents the thermal effects and damage caused by thermal diffusion and creates a neat and precise processed edge [41,42].

### 2.3. Laser-Induced Plasma

The laser processing process is significantly impacted by the plasma produced during the laser processing. Plasma, also known as the fourth state of matter, is mostly made up of atoms, molecules, electrons, free radicals, and different reactive groups. When irradiated by a high-energy density laser, CFRP produces partially ionized plasma. Under the influence of plasma, more oxygen and nitrogen polar groups are produced on the surface of CFRP, and the interfacial bonding strength is greatly increased [43]. However, the plasma cluster concentrating at the processing site may reduce the target’s ability to absorb laser energy, lowering processing effectiveness and ablation quality [44]. Besides, the plasma eruption and plume smoke in CFRP laser processing cannot be effectively predicted by temperature-field simulation. To further explore the removal mechanism of CFRP, researchers use high-speed cameras to directly record the laser ablation process of CFRP.

**Figure 7:**
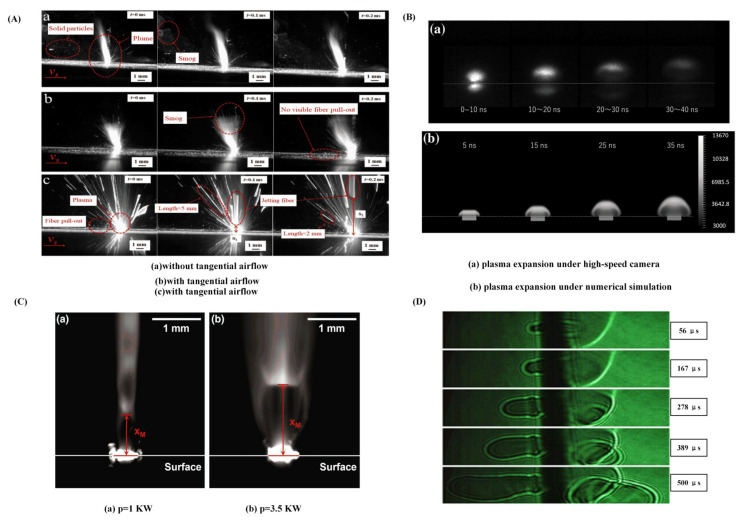
Plasma expansion of laser-processed CFRP captured by high-speed camera: (**A**) plasma in various air ambiances [36]; (**B**) burning waves at various pulse counts [47]; (**C**) diagram of a plasma plume in smoke [46]; (**D**) the phenomena of mushroom-like eruptions [45].

Figure 7A illustrates the results of CFRP laser ablation studies carried out by Tao et al. [36] in both air and vacuum. Small solid particles floating on the surface of the workpiece and the plasma mixture will be swept away by the tangential airflow. Ma et al. [45] investigated the impact of the plasma produced during laser manufacturing on the damage process and recorded the plasma plume by a high-speed camera, as shown in Figure 7D. It was found that the formation plasma and the temperature on the surface of the material was related to the change in pulse number. Faas et al. [46] used high-speed imaging technology to analyze the thermal plume of the ablation products created during CFRP laser drilling, as shown in Figure 7C. It was found that compression waves had formed in the flowing evaporated material, and the flow rate of the thermal ablation products could be estimated by examining the location of these compression shocks. Ohkubo et al. [47] noticed mushroom-like eruptions during laser processing CFRP using a high-speed camera. Meanwhile, they performed numerical simulations to study the dynamics of the processing process, as shown in Figure 7B. According to the findings, the degradation of the resin material is significant at low processing depths, and the thermal influence is more severe at high processing depths. Bluemel et al. [48] investigated CFRP laser processing under various process conditions with a 1.5 kW fiber nanosecond-pulsed laser. The plasma and plume smoke produced in laser processing CFRP were analyzed, showing that the laser-induced plasma lifetime ranged from 20 to 200 ns.

**Figure 8:**
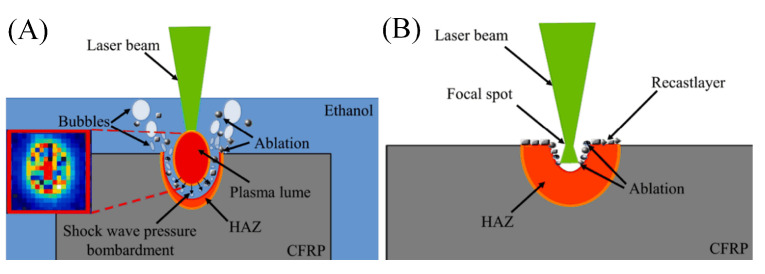
Laser-induced plasma micro-drilling method versus conventional laser processing: (**A**) schematic of LIPMD process; (**B**) schematic of LIA process [49].

With the further investigation of the plasma’s mechanism, Wang et al. [49] proposed the use of laser-induced plasma micro-drilling to reduce taper and improve surface integrity after processing. As shown in Figure 8A, it can effectively enhance the quality and roundness of micro-hole edge. Under the same parameters, the photo-induced plasma micro-drilling method reduced the hole taper by 32.02% compared to the traditional method, as shown in Figure 8B.

In conclusion, the laser processing process is accompanied by many intricate physical effects, including photothermal, photochemical, and photomechanical effects. The action mechanism of the laser on CFRP mainly depends on the wavelength and pulse width. In addition, variations in pulse width and wavelength are expected to alter the relative importance of numerous effects. The proper use of plasma can enhance processing quality and interface bonding strength, but plasma processing is a significant challenge in laser processing research. The current analysis of the dynamic observation results of the laser processing process mostly uses high-speed cameras to capture the plasma effect in the laser processing process.

## 3. Main Factors Affecting the Laser Processing of CFRP Materials

### 3.1. Laser Wavelength

The amount of laser energy that is absorbed by the CFRP when it is exposed to laser radiation dictates how much of the total laser energy is used. Epoxy resin is employed as the material matrix, and the epoxy resin’s absorption property of the laser defines the first laser action process. The laser action process on the carbon fiber composite material is closely related to the laser wavelength. The removal mechanism of the action process is determined by the energy of the single laser photon, which increases as the wavelength decreases.

**Figure 9:**
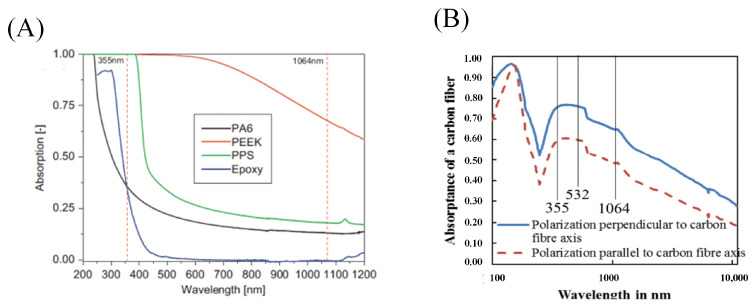
Absorption rates of different wavelengths of light by carbon fiber and epoxy resin respectively: (**A**) absorption spectra of resin materials [50]; (**B**) the average absorption curves of single carbon fiber for different laser wavelengths [51].

Romoli et al. [50] measured the absorption rates of polymers as the matrix to lasers at different wavelength by the spectrophotometer, as illustrated in Figure 9A. The rate of absorption increases with decreasing wavelength. When the wavelength is longer than 650 nm, the resin can hardly absorb the laser. Friedrich et al. [51] investigated the effect of a polarized laser on the interaction between laser and carbon fibers. Based on the Fresnel equation, a theoretical model was used to determine that the absorption of carbon fiber textiles and CFRP decreases with increasing wavelength. Figure 9B shows the absorption of a polarized laser at different wavelengths by carbon fibers. For the vertically incident laser beam, the absorption rate in the direction of polarization parallel to the carbon fiber is slightly lower than that of the polarization direction perpendicular to the carbon fiber.

The absorption rate of CFRP to laser decreases with the increase in laser wavelength, and the smaller the spot diameter and higher the laser power, the more significant the absorption rate fluctuation is. Besides, the volume fraction of randomly distributed carbon fibers also influences the absorption rate. The bulk absorption of the material decreases with increasing material thickness. The laser wavelength of 10.6 µm is the only wavelength that has higher absorption performance in epoxy materials than in composites or carbon fibers [52,53].

In the laser processing process, the laser wavelength not only affects the rate of CFRP absorption, but also significantly affects the HAZ in the material. The HAZ created during the laser processing shrinks as the wavelength is shrunk. Wolynski et al. [54] processed CFRP by laser in wavelengths of 1064 nm, 532 nm, and 355 nm, respectively. The results showed that the HAZ produced by laser cutting with the 1064 nm wavelength was twice as large as that produced by cutting with a 532 nm wavelength. Dell’erba et al. [55] demonstrated that there is no heat transfer between the excimer laser and the composite material, and excellent cutting quality is obtained. However, the processing efficiency of the excimer laser is incredibly low, making it unsuitable for industrial applications. As a result, excimer wavelength lasers are used less frequently for laser processing CFRP to ensure processing efficiency. Table 1 summarizes the research on the size of the HAZ by wavelength in recent years, and it can be seen that the heat-affected zone shrinks as the wavelength decreases.

The absorption rate of CFRP to laser increases as the wavelength decreases and the energy of individual photons also increases. When the laser wavelength is ultraviolet, photochemical processing can greatly reduce thermal damage and improve processing quality, so the heat-affected zone range decreases with the wavelength. Short-wavelength lasers should therefore be chosen to increase energy efficiency and decrease material damage.

### 3.2. Laser Pulse Width

Since there is a time interval between each pair of pulses, the material can cool more easily and sustain less thermal damage in processing than by using a continuous laser. Because the laser power is certain and a pulsed laser is discontinuous in time, the shorter the laser pulse width, the higher the energy density of a single pulse, which is favorable to quick material removal and reduces HAZ production. Compared to millisecond pulses, nanosecond pulses have a smaller HAZ and produce superior processing outcomes [57,58].

In contrast to traditional long-pulse laser processing, ultrashort-pulse laser processing instantly ablates and strips off the processed material instead of heating, melting, and vaporizing. This eliminates the cooling recasting process and enables ultra-fine processing. There are several benefits of ultrafast laser “cold processing”, including low temperature effects, exact edges at the tiniest scales, no material constraints, and no alterations to the mechanical, chemical, physical, or structural characteristics of the surrounding medium. Due to the obvious thermal effect of processing, traditional lasers like continuous lasers, millisecond, and nanosecond form HAZ with a scale of 100 to 2000 μm, whereas ultrashort-pulsed lasers like picosecond and femtosecond lasers can achieve high-quality cutting with a HAZ width of only 5 to 100 μm [59,60,61,62,63].

The typical power of picosecond and femtosecond ultrafast lasers is now between a few tens and hundreds of watts, which means that the application is still dominated by laser microfabrication. This contrasts with continuous lasers, which have an average power of 10 kW. The average power of industrialized ultrashort-pulse laser technology has increased significantly in recent years from 10 watts to several kilowatts. Ultrafast laser processing technology is anticipated to make significant advancements in processing efficiency, thickness, and area, while also maintaining the advantages in processing accuracy and quality currently enjoyed by this technology [64,65].

The effect of cold processing can be obtained when the laser pulse width achieves picosecond. Continued pulse width reduction will not only result in a decrease in processing efficiency but will also have a negligible impact on reducing the heat-affected zone. In reality, the advantage of “reducing the time of laser-material interaction” of an ultrafast laser can be offset by the heat accumulation effect caused by unsuitable parameters. Therefore, HAZ should be reduced by optimizing the laser processing parameters and processing methods to improve processing quality in actual experiments [66,67]. Table 2 provides a summary of the recent research on the effect of pulse width. As can be seen, the HAZ gets smaller as the pulse width gets smaller.

**Figure 10:**
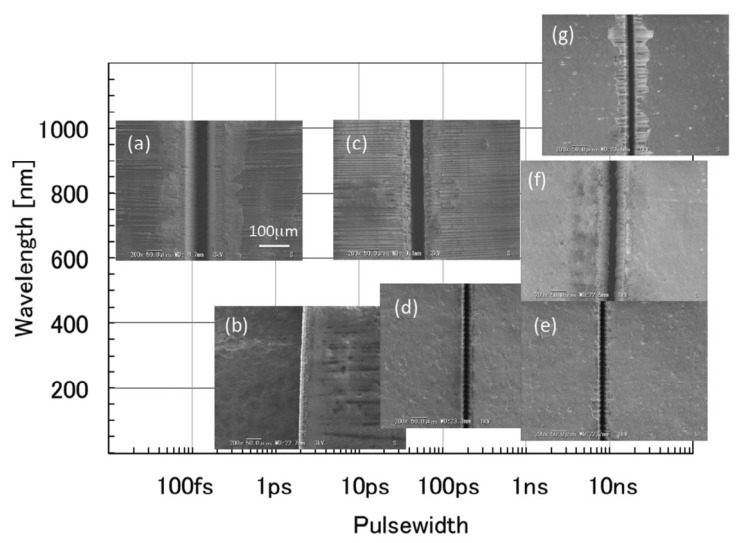
Processing morphology with different wavelengths and pulse widths, (**a**) the pulse width and wavelength of the laser beam of-(**g**) are respectively: (**a**) 180 fs, 800 nm; (**b**) 35 ps, 800 nm; (**c**) 200 ps, 800 nm; (**d**) 2 ns, 355 nm; (**e**) 10 ns, 355 nm; (**f**) 10 ns, 532 nm; (**g**) 20 ns, 1064 nm [68].

Fujita et al. [68] investigated the variation of HAZ in CFRP and cutting efficiency under various laser cuttings from 266 nm to 1064 nm and laser pulse widths from femtosecond to nanosecond, as shown in Figure 10. This further confirmed that a shorter laser wavelength and pulse width could achieve smaller HAZ, as well as higher cutting efficiency.

Therefore, using shorter wavelengths and ultrashort pulse processing can significantly reduce the heat-affected zone to improve the quality of the processing. In conclusion, nanosecond lasers, continuous lasers, and other traditional lasers form the HAZ scale in the range of 70 μm to 1000 μm. However, picosecond lasers, femtosecond lasers, and other ultrafast lasers can achieve HAZ widths of only 5 μm to 100 μm. However, the production of thermal effects is also strongly influenced by the laser processing process parameters. Experiments are carried out to adjust the process parameters during the interaction between the laser and material to get a superior processing quality.

### 3.3. Scanning and Focusing Parameters

The development of HAZ is also strongly influenced by the laser processing process parameters. A small HAZ can be achieved by adjusting the laser settings. However, unsuitable laser parameters could exponentially expand the HAZ. To improve the processing quality of CFRP components, laser process parameter adjustment is crucial.

**Figure 11:**
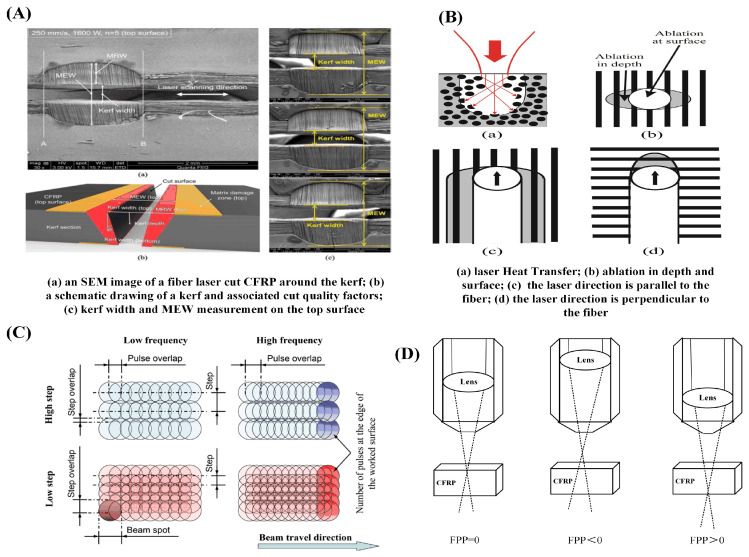
Schematic diagram of the effect of laser process parameters on the processing of CFRP in laser processing: (**A**) effect of laser processing parameters on CFRP [69]; (**B**) schematic diagram of the different scanning directions of the laser [73]; (**C**) graph of scanning speed versus repetition frequency [37]; (**D**) different out-of-focus situations for laser processing.

Oh et al. [69] proposed the factors affecting cutting quality through the experiment of the 2 KW fiber laser cutting CFRP, and described the laser processing cutting quality, as shown in Figure 11A. The experiment confirmed that the scanning speed is the dominant factor affecting thermal damage, and increasing the cutting speed can reduce thermal damage. Takahashi et al. [70] performed an experimental investigation on cutting CFRP with an infrared nanosecond laser. The experiments were carried out utilizing a scanning oscillator to adjust the processing position. It was claimed that raising a specific scanning spacing may lessen the vapor shielding effect, which could result in a large increase in processing speed and improve processing quality.

The diverse carbon fiber lay-ups used in CFRP, as well as the various scanning orientations used during laser processing, have a big impact on the laser processing. Fiber lay-up and laser scanning directions are shown in Figure 11B. The area of HAZ is the largest when the laser scanning direction is perpendicular to the surface fiber lay-up direction, and it is least when the scanning direction is synchronized with the surface fiber lay-up direction [71,72,73]. When laser cutting double-layered CFRP sheets with different arrangements, the cutting of double-layered CFRP consisting of 90° or 45° with 0° orientation can get a better quality of cut [74,75]. The direction of the fibers has a significant impact on the way carbon fiber composite laminates are damaged. Laser scanning path planning can keep the angle at 45°, which is helpful for ensuring processing quality and increasing efficiency.

The following laser processing CFRP parameters have the most impact on the heat-affected zone: laser power, scanning speed, repetition frequency, defocusing, and others.

The laser power directly affects the amount of laser energy absorbed by the material per unit of time. Excessive laser power will cause the processed material to burn excessively, whereas too low laser power may lead to the nonpenetration of the material, requiring repeated processing. This will affect processing efficiency and lead to excessive heat accumulation, resulting in the expansion of the heat-affected zone [76,77,78].

In laser processing, the scanning speed determines the time for the laser to interact with the material. When the scanning speed is too high, the laser beam acts on the material for a short time, the heat conduction and heat diffusion are insufficient, and the processed material cannot be cut through because of insufficient heat input [77]. The pulsed laser behaves as a continuous pulse at a higher repetition frequency and slower scanning speed, causing excessive ablation in material, resulting in an excessive region of a heat-affected zone and reduced cutting quality [37]. The relationship between the scanning process parameters is depicted in Figure 11C.

To obtain a better processing effect, the laser beam converging on the surface of the workpiece surface is focused into a point, just landing on the surface of the material to be processed, that is, zero defocus, to get a superior processing result. The power density is at its highest at this moment [79]. For positive defocusing, the laser’s focal plane is above the surface of the processed workpiece; conversely, for negative defocusing, the laser’s focal plane is below the treated workpiece. Different focal length scenarios, as shown in Figure 11D, will result in energy diffusion and a reduction in the processing quality for both positive and negative out-of-focus [80].

Thermal damage is unavoidable when the laser interacts with CFRP. Selecting suitable process parameters can realize the low-damage laser processing of CFRP to manage the laser–material contact time and the energy irradiated into the material. However, each parameter in the laser processing process is not simply combined. Due to differences in laser-spot diameters, material thicknesses, material properties, and laser type, the relationship between each parameter needs to be considered to obtain the best processing results.

### 3.4. Laser Scan Path

**Figure 12:**
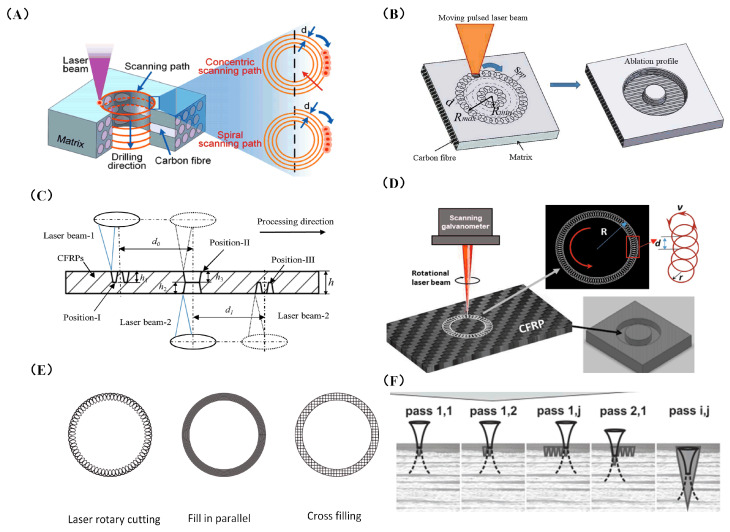
Various scanning techniques to improve quality and efficiency in laser processing: (**A**) schematic showing two distinct scanning pathways and the laser-drilling procedure [82]; (**B**) pulsed-laser coaxial-trepan drilling technique illustrated schematically [84]; (**C**) schematic diagram of dual-beam opposite dislocation (DBOD) laser-drilling process [84]; (**D**) the picosecond laser “double rotation” cutting method [87]; (**E**) schematic diagram of scanning mode [88]; (**F**) the approaches used in the experiments to define the maximum possible cutting depth [89].

The thermal effect of the laser easily generates a HAZ in the material, but the material removal is difficult to control in the depth direction. The processing depth–diameter ratio is too large, or the scanning method is improperly selected, which can easily cause uneven material removal and reduce processing accuracy. If only relying on laser vaporization ablation, the material removal efficiency is also low. Therefore, several researchers have optimized the laser processing process, which not only reduces the HAZ, but also improves the processing depth and efficiency. Li et al. [81] proposed a coaxial ring hole-making procedure. First, the outline is sliced, then gradually smaller and smaller circles are cut inward. The laser beam motion trajectory consists of a series of concentric rings, and the distance between concentric rings is generally set to a focused-spot diameter, which is conducive to the complete removal of material from the target area. The circular path after processing has enough cooling time, which reduces the generation of the HAZ. Li et al. [82] investigated the scanning modes based on the multi-pass method, including the concentric scanning mode and the spiral scanning mode, as depicted in Figure 12A. It was found that the spiral scanning mode can significantly reduce thermal damage. The diameter of the HAZ fell from 127.3 μm to 84.76 μm. In the same year, they proposed a staggered scanning processing mode, which facilitates better material removal during drilling and the timely discharge of broken fibers and vapors, and reduces the degree of thermal decomposition of the frontier matrix material and the thermal accumulation effect of adjacent trajectories. As a result, the average width of HAZ was reduced overall by 25.85%, and the taper was also reduced by 51.45% in holes with the same parameters [83]. Tao et al. [84] proposed improvements based on the method of making holes in the coaxial ring, Figure 12B is the coaxial ring hole making procedure, they suggested a dual-beam laser hole-making procedure for thick CFRP plates. As illustrated in Figure 12C, two pulsed laser beams positioned in opposite positions are used to simultaneously cut holes in CFRP. The laser beam scanning path is carried out in a coaxial ring. In this process, the effective removal depth of a single laser is greatly increased, which greatly improves the processing efficiency. Meanwhile, this process suppresses the problems of low processing efficiency and large HAZ damage caused by the blocking of the laser beam because of the increase in depth. A novel stepped-parameter parallel-ring laser-drilling technique was developed by Zhu et al. [85] in which the laser beam starts drilling from the outer to the inner ring. This method increases the energy input of the inner ring, enabling the removal of material more quickly, while the lower energy input of the outer ring provides a shielding trench to reduce heat loss from the parent material. With the help of this procedure, quality and productivity have increased by over 300%, energy consumption has decreased by 78.10%, and CO_2_ emissions have decreased by more than 25%.

Fornaroli et al. [86] proposed a circular motion of the laser beam around the center of the target hole while the beam axis rotates around its own center. The laser beam’s circumferential path rotation is synced with the beam’s internal rotation and spirals into the process. A through-hole is created by repeatedly abrading the target hole material with laser pulses. It significantly improves the HZA and hole taper. In the study of Ouyang et al. [87], the CFRP was processed by the picosecond laser in a “double rotation” cutting technique, as shown in Figure 12D. Compared to mechanical processing, the mechanical properties of the processed CFRP samples, such as tensile strength and fatigue parameters, were enhanced. Additionally, the drilling precision, taper, and HAZ are all under good control. Ye et al. [88] investigated the effect of different cutting techniques on the accuracy of CFRP hole making. As shown in Figure 12E, laser rotary cutting produces holes of the highest quality compared to parallel and cross-cutting techniques. Herzog et al. [89] suggested a parallel channel to broaden the kerf, as shown in Figure 12F. CFRP can be sliced up to 13 mm thick by focusing the laser into the edge of the notch. These scanning methods were compared, as shown in Table 3.

### 3.5. Processing Environment and Auxiliary Agents

**Figure 13:**
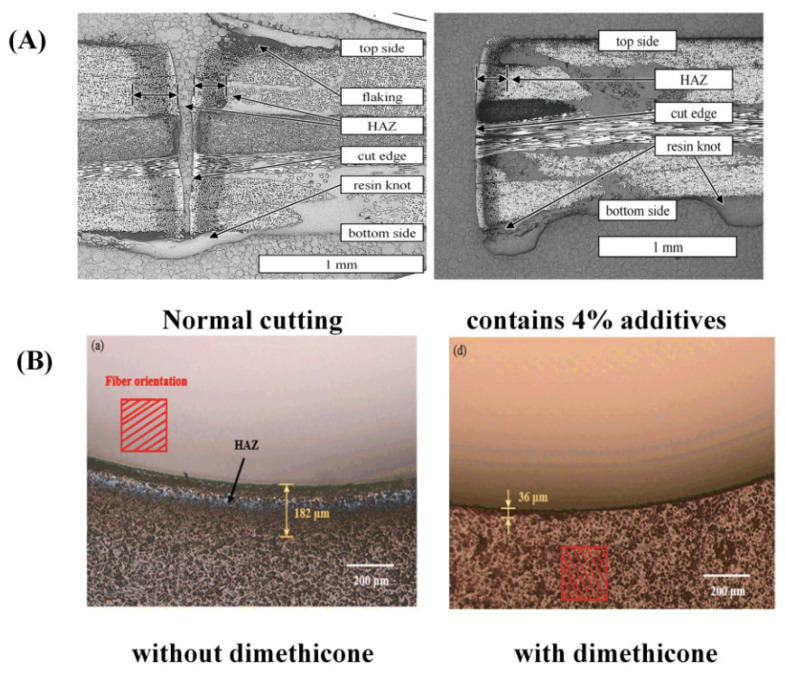
The effect of auxiliary agents on laser processing: (**A**) CFRP morphology after carbon-black-assisted laser processing and conventional laser processing [94]; (**B**) CFRP morphology after dimethylidene-ketone-assisted laser processing and conventional laser processing [95].

Due to the large difference between the properties of the matrix and the reinforcing phase in CFRP, the auxiliary agents are added to CFRP to balance the difference between these two parts, which reduces heat diffusion and secondary damage during laser processing. According to the study of Staeh et al. [90], when laser processing of CFRP was performed under various humidity processing settings, with the increase in material humidity, the shear modulus of material decreased and the diameter of HZA rose as the moisture content of the material increased. Jaeschke et al. [91] discovered that the HAZ is more prone to absorb moisture from the surroundings when cutting CFRP with a CO_2_ laser, leading to secondary damage and weakening the mechanical properties of the material. Therefore, to decrease moisture absorption and enhance the surface quality of CFRP, polyamide powder was injected using a nozzle into the treated surface to fill in the gaps created after the resin vaporized. The addition of a 1.5% mass fraction of multi-walled carbon nanotubes as secondary reinforcement to the polymer matrix lowers the difference in decomposition temperature between carbon fiber and epoxy resin, and the high thermal conductivity of multi-walled carbon nanotubes raises the thermal diffusion coefficient of the polymer matrix [92]. In comparison to the processing results of traditional CFRP, the experiments demonstrate reductions in HAZ, taper angle, and surface roughness of the hole of around 30%, 47%, and 43%, respectively. Canisius et al. [93] suggested that applying a laser absorption additive (carbon black) to boost the absorption rate of the resin can greatly lower the HAZ. Figure 13A illustrates that the HAZ can be reduced by 25% by adding 4% carbon black to the material. Additionally, experimental results showed that adding carbon black particles to the resin matrix might enhance cutting and lessen the production of processing flaws [94]. The dimethyl ketone-aided laser-cutting method was employed by Chen et al. [95]. As illustrated in Figure 13B, the HAZ width was decreased to 38.70 μm with dimethyl-ketone-assisted processing as opposed to 274.37 μm with straight laser processing, a reduction of 85.89%. This is because dimethyl ketone’s heat transfer inhibition prevented practically all substrate material combustion.

## 4. New Laser Processing Process Methods

Researchers have suggested measures to decrease the size of the processing heat-affected zone, including cooling the processing area by convection from the surrounding environment, lowering the temperature at which thermal damage occurs, and increasing processing efficiency. For example, these measures include using airflow to remove some of the heat and plasma gas to increase processing efficiency and depth of cut, as well as using water’s scouring and cooling effects to lower thermal damage.

### 4.1. Gas-Compound-Assisted Laser Processing

Laser processing with gas assistance, different gas types, air pressure, optical gas coaxial, or side axis assistance will bring different processing impacts.

**Figure 14:**
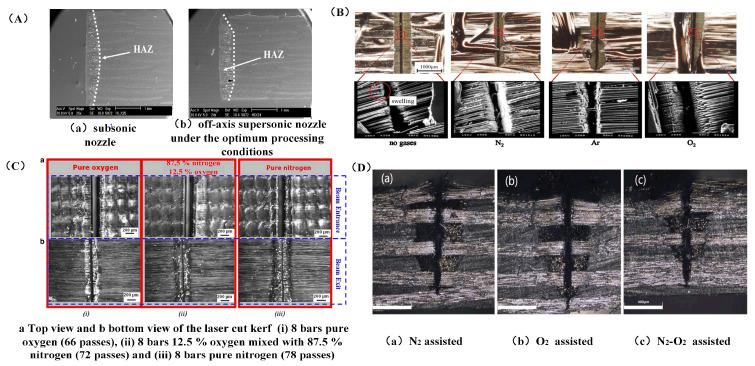
Effect of different auxiliary gases on laser processing of CFRP: (**A**) two different kinds of nozzle processing [97]; (**B**) cross-section view of different gas processing CFRP sample processes [98]; (**C**) the effect of gas processing with different contents [99]; (**D**) different gas processing [100].

Zhang et al. [96] conducted a comparison test of laser irradiation damage of carbon fiber composite materials on the subsonic flow and no-flow environment. According to the test results, the processing area had a certain cooling effect when the tangential air flow was assisted. As the pressure increased, the gas flow increased, which can remove more heat for cooling and reduce the heat-affected zone. Riveiro et al. [97] studied the assisted-laser cutting experiment of Ar gas at different air pressures with a 3.5 KW CO_2_ laser. Experiments showed that gas sprayed through coaxial subsonic nozzles and paraxial supersonic nozzles can blow away heat better, thus reducing the heat-affected zone. The morphology was shown in Figure 14A. Yuki et al. [98] used nitrogen, argon, and oxygen as auxiliary gases to study laser processing under the same average power and scanning speed, and the results are shown in Figure 14B. Because the specific heat capacity of nitrogen is twice as large as that of argon, it can absorb more heat and suppress the heat-affected zone. Therefore, nitrogen and argon as auxiliary gases can eliminate the expansion of the carbon fiber end face. With the help of oxygen, the maximum depth of incision may be accomplished, solving the issue of challenging material removal in the deeper layers of the material because of lower laser energy.

Negarestani et al. [99] used a nanosecond Nd:YAG laser to process CFRP; pure nitrogen, pure oxygen, and a nitrogen–oxygen mixture were respectively used as auxiliary gases for processing. The cutting morphology under the three gas-assisted modes is shown in Figure 14C. Under the aid of the nitrogen and oxygen mixture, the pull-out of bottom fibers was increased by 47% and 59%, respectively. In the meantime, inert N_2_ may rapidly chill the processing region, minimizing thermal damage, while active O_2_ can improve the material removal rate and accelerate the disintegration of composites by exothermic reaction. For the laser cutting of carbon-fiber-reinforced plastics, Qin et al. [100] used three gas-assisted techniques: coaxial nitrogen, coaxial oxygen, and coaxial oxygen near-axial nitrogen composite gas to assist. The nitrogen–oxygen mixture had the greatest etching depth, up to 1000 μm, and the nitrogen cooling effect was better. The dual gas flow further improved the blowing impact on the melt, which gave the gas mixture the benefit of cooling and enhancing etching. Figure 14 displays the three distinct gases’ processing effects (D).

In conclusion, the experiment revealed that oxygen enhances the decomposition process of composites through exothermic reaction, improves the material removal rate, and solves the issue of the challenging material removal caused by the reduction in laser energy in deep layers of materials. Inert gas has a certain inhibitory effect on the heat-affected zone of carbon fiber composites. Therefore, a nitrogen–oxygen composite can be used to provide the best processing result.

### 4.2. Liquid Composite Auxiliary Processing

In 1842, Colladon demonstrated the possibility that water jets could guide light by discovering that light could travel along the curve of a water jet. Then in 1854, Tyndall discovered the phenomenon of total reflection of light in flowing liquid through experiments and confirmed that water jets could be used as optical fibers to transmit light. In 1987, Doi attempted to fuse lasers with water to form “laser knives” that could apply laser radiation to a processed surface. In 1990, Wrobel et al. [101] successfully connected a jet to a solid fiber to direct a laser to the workpiece surface. Since then, the research on water as a liquid assisting in laser processing has been started. The processing methods of a liquid-assisted laser can be divided into underwater-assisted processing, water-jet-assisted processing, water-guided processing, etc. The cooling effect of flowing water is more obvious than that of gas, and water can scour the residue to obtain a clean processing surface. However, due to the better heat-absorbing effect of water, the laser energy loss is also relatively large.

Hua et al. [102] used a 500 W millisecond pulse Nd:YAG laser to process carbon fiber composite materials. It was found that the thermal damage of laser cutting was greatly reduced, and the heat-affected zone and fiber pulsing were reduced when cutting underwater. Tangwarodomnukun et al. [103] compared and analyzed the width of the heat-affected zone in laser processing of CFRP in air, still water, and running water. It was found that the heat-affected zone produced by processing in both still water and running water can be significantly reduced.

**Figure 15:**
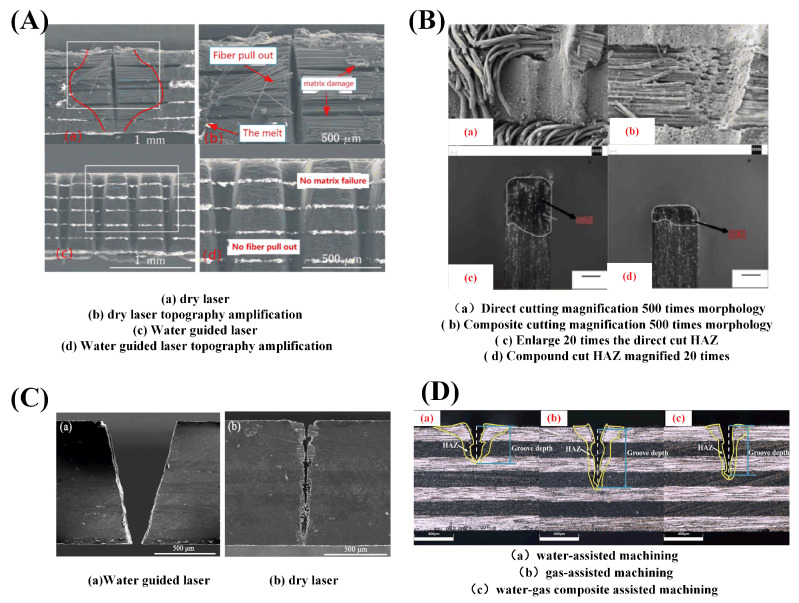
Schematic diagram of the morphology of CFRP processed by liquid-assisted laser: (**A**) comparison of the visual effects of dry laser cutting versus water-jet-assisted laser cutting [104]; (**B**) water-jet-assisted laser light topography [105]; (**C**) dry laser processing and water-guided processing [107]; (**D**) morphology of three auxiliary processing modes [110].

Wang et al. [104] processed CFRP using a water-guided laser. The research results showed that the cutting surface of CFRP processed by a water-guided laser was clean and flat. In addition, there were almost no fused impurities attached to the cutting surface and groove, as shown in Figure 15A. Additionally, the surface heat-affected zone, material delamination phenomenon, and fiber expansion were all improved. The cooling effect of the paraxial water jet was exploited by Zhang et al. [105] to restrict the diffusion of excess heat produced during laser processing. As can be seen from the morphology of Figure 15, the smaller the heat-affected zone was, the more water was flowing through the system (B). Water-jet assist can not only achieve timely heat dissipation but also improve the cutting surface processing quality of carbon fiber and reduce carbonization.

**Figure 16:**
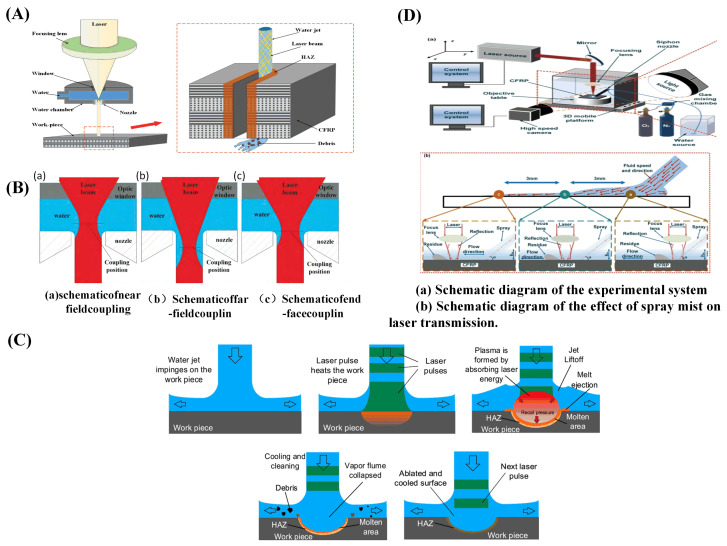
Liquid-assisted laser processing CFRP method and water-guided laser processing CFRP technology principle: (**A**) schematic for cutting CFRP with a laser guided by a water jet [106]; (**B**) there are several ways to combine water jets and light [108]; (**C**) a typical water-jet-guided laser ablation cycle [109]; (**D**) flowchart of the thin-water-film-assisted laser processing method [111].

Sun et al. [106] cut carbon fiber composite materials with a water-guided laser. During water-guided laser processing, the heat-affected zone can be greatly reduced and the processing quality can be improved. Figure 16A shows the principle of water-guided processing. In the same year, they compared conventional laser and water-guided laser processing of carbon fiber composites. As shown in Figure 15C, the average size of the heat-affected zone in the traditional laser machining was 37 μm, while there was almost no obvious damage to the substrate of the cutting surface in the water-guided laser cutting, but the cutting speed was slower, only one half of that in traditional laser machining [107].

For water-guided lasers, Wang et al. [108] showed that the water’s absorption coefficient of light drops as the wavelength decays. The nozzle construction is emphasized as having a significant impact on the jet flow parameters, including jet pressure, flow rate, and velocity and determines whether a stable “reduced flow” water jet can develop. Additionally, the examination of the coupling revealed that there are three different types of coupling: end-face coupling, far-field coupling, and near-field coupling, as depicted in Figure 16B. According to Liu et al.’s [109] summary of a water-jet-guided laser ablation cycle depicted in Figure 16C, cutting precision increases with decreasing water-jet diameter, while water-jet stabilization becomes more challenging. When drilling a deep hole, water becomes trapped inside the hole; this water turbulence and the leftover material inside the hole can influence laser propagation. The processing speed of deep holes was impacted by the high power density of the laser beam energy in the water at the same time. Three alternative cooling techniques—water, gas, and a water–gas composite—were compared by Zhang et al. [110] in their comparative research of carbon fiber composites processed with laser assistance. A water and gas compound assisted laser processing, blowing away the water in the laser irradiation area, greatly reducing the interference of the water on the laser transmission, making the laser more concentrated in its irradiation of the material, while the impact of the gas on the water will make the water into smaller droplets or even cause atomization, which will make the water easier to absorb heat-phase change and vaporization, combined with nitrogen cooling, it further reduces the residual heat generated during the processing process. Figure 15D is a schematic representation of several processing scenarios. To help the laser in treating CFRP’s low-temperature damage, in the same year, the team used a spray impingement on the tube wall to create a thin flowing water film. A mixture of nitrogen and oxygen was employed to help the siphon nozzle create a spray mist based on the oxidation process of oxygen to enhance the etching efficiency of CFRP, as illustrated in Figure 16D. Additionally, it was discovered that a high processing quality could be produced at a 30% oxygen concentration and a relative balance of heat and cold [111].

In conclusion, water-jet-assisted machining can cool the edge of the cut formed by a laser pulse on the material surface and the surrounding area. Therefore, HAZ and thermal residual stress were reduced to prevent thermal damage inside the material. High-precision and high-quality ablative and cutting edges can be achieved using water-guided laser machining. Compared with dry laser cutting, the kinetic energy of any auxiliary gas flow used in liquid laser cutting was much higher. The water jet can discharge the molten material more effectively, and the ablative products combine with water, which can greatly reduce the release of harmful substances into the atmosphere. However, this kind of laser energy loss was relatively large and processing efficiency was low.

### 4.3. Laser Composite Processing Method

The physical process of laser interaction with materials was very complex, and the processing method of a single traditional laser beam solved the problems of low cutting thickness and low processing efficiency with difficulty. The CFRP matrix easily absorbed water and dehydrate; wading processing is generally prohibited in the field of formal aviation product processing. To improve the quality and efficiency of laser processing in traditional dry laser processing, a new processing method has been proposed.

**Figure 17:**
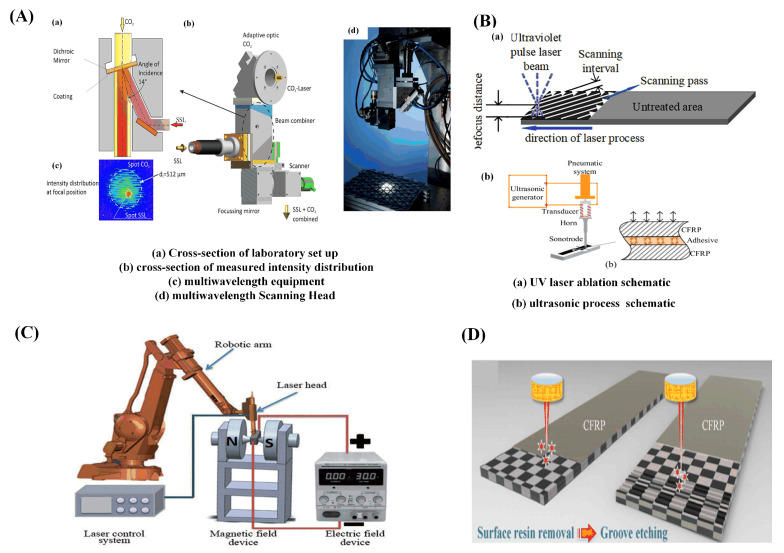
Schematic diagram of the new processing method of laser processing: (**A**) schematic diagram of dual-wavelength combined laser [112]; (**B**) the schematic diagram of the two-step laser surface treatment of a CFRP laminate [118]; (**C**) schematic of electromagnetic-field-assisted laser groove processing device [117]; (**D**) ultrasonic and laser process schematic [116].

Furst et al. [112,113] used a laser with a power of 1 kw composed of a carbon dioxide laser with a wavelength of 10.6 μm and a fiber laser with a wavelength of 10.6 μm to cut carbon fiber composites, in which CO_2_ accounted for 25% of the total power and the fiber laser accounted for 75%. The experimental principle is shown in Figure 17A. In this way, cutting quality and cutting depth were improved.

Jia et al. [114] aimed at the problems existing in the processing of CFRP materials, proposed the combined laser and mechanical processing method. First, a high-power laser was used for rough machining to remove a large amount of material allowance. Then, mechanical processing was used to remove this part of material to improve the processing quality and dimensional accuracy of the final component. On this basis, Chen et al. [115] proposed a collaborative fiber-laser–numerical-control milling processing of a 10.0 mm thick CFRP plate, which integrated the advantages of high efficiency, high material removal rate, good surface quality, and high cutting speed in the high-power laser cutting process and improved the processing quality and speed. Xie et al. [116] used a two-step laser surface treatment to improve the performance of single-loop bonded joints in CFRP-laminates, as shown in Figure 17D. In the first step, the resin is completely removed from the outer surface of the laminate by an optimal laser-etching process; this provides a neat carbon fiber fabric. In the second step, the resulting exposed carbon fiber fabric is further irradiated to produce a series of small grooves on its surface.

Hu et al. [117] proposed an electromagnetic compound field-assisted laser removal technique for the groove forming of materials. Compared to traditional laser material removal methods such as laser punching and laser cutting, this method was able to perform the processing of blind grooves efficiently and continuously, and the experimental setup is shown in Figure 17C. The principle was as follows: under the control of the manipulator, the high-energy laser beam was irradiated on the surface of the workpiece material, and the irradiation area became a molten state. The external steady-state magnetic field and electric field will generate a Lorentz force in a directional direction. When the Lorentz force was greater than the sum of the gravity and surface tension of the material in the molten area, the molten material in the molten area will be discharged through the upper part of the groove. Thereby achieving the effect of electromagnetic composite field assisted laser material removal and groove forming.

Wang et al. [118] processed CFRP by combining both laser and ultrasonic vibration, as shown in Figure 17B. Initially, the laser ablates to remove the resin, exposing the fibers and creating ordered grooves on their surfaces. Subsequently, the CFRP laminate was subjected to ultrasonic vibration during the bonding process to facilitate penetration of the adhesive into the micro-scale groove structure. In this way, the shear strength of CFRP was increased by 340%. Zhou et al. [119] proposed a method for ultrasonic-vibration-assisted laser processing in ethanol solutions to reduce the adverse effects of cavitation bubbles on the processing. HAZ and etch depth were reduced by 57% and 25%, respectively. Ultrasonic vibration explodes the cavitation bubble and reduces the interference of the cavitation bubble with the laser, the effect of mechanical erosion was enhanced, and the etch depth is increased by 119%. Ultrasonic vibration enhances the cooling effect in the ablation region, and HAZ was reduced by 57%.

### 4.4. Laser Surface Cleaning Technology

CFRP has many advantages, but it also has disadvantages such as low compressive strength and low temperature resistance. In most applications, CFRP needs to be combined with metal to build the complete structure. Traditional mechanical connections (e.g., riveting and bolting) between CFRP and metal joints reduce the static strength of the joint, and laser connection technology has become a very fast developing connection technology in recent years, ideal for CFRP-to-metal connections because of its low heat input, high precision, and high efficiency. In laser connection technology, the laser acts in two different ways. First, due to the difference in properties of epoxy resin and carbon fiber, to ensure or enhance the interface connection strength and durability through the laser cleaning method, physical cleaning of resin, increasing effective joint area, and chemical activation are achieved. The main connection mechanism is mechanical interlock connection [120,121].

**Figure 18:**
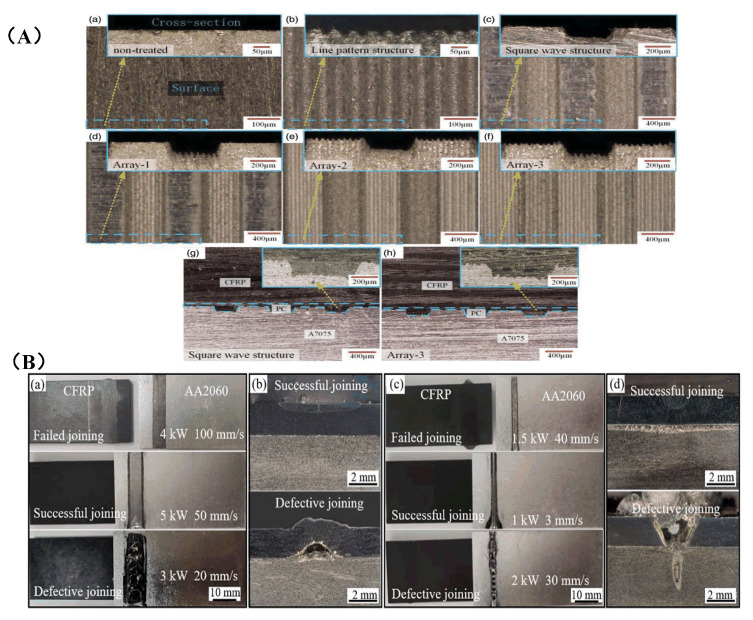
Cross-sectional and surface pictures produced by laser processing: (**A**) (**a**–**f**) six different scan arrays; (**g**,**h**) cross-sectional images of Al-PCCFRP adhesion lap-joint samples with interfacial microstructures: the square wave structure and array 3 [126]; (**B**) typical surface and cross-sectional morphology of CFRP/AA2060 joints: (**a**,**b**) rectangular and (**c**,**d**) circular spots [131].

**Figure 19:**
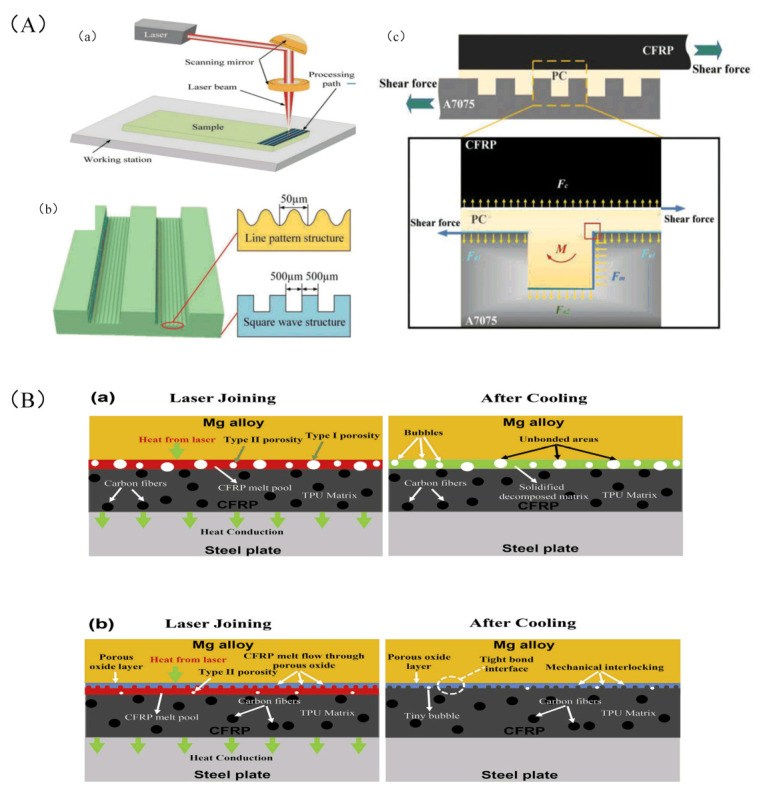
Two different principles of laser cleaning of CFRP: (**A**) A7075 sheet laser surface treatment; (**a**) schematic diagram of laser surface treatment of A7075 plate; (**b**) schematic diagram of two different microscopic structures; (**c**) force analysis diagram [126]; (**B**) schematic diagrams illustrating the mechanisms involved in the LAMP joining between the CFRP and AZ31 Mg alloy; (**a**) CFRP and as-received AZ31 Mg alloy; (**b**) CFRP and thermally oxidized AZ31 Mg alloy [130].

Oliveira et al. [122,123] experimentally studied the effect of femtosecond laser processing on the micro/nano structure of a CFRP surface. The selective removal of epoxy resins is presented and leaves exposed carbon fibers with the desired pattern. In addition, a submicron-laser-induced periodic surface structure is generated on the carbon fiber surface, which helps to improve the adhesion of the fiber to the substrate in the adhesion bonds between the CFRP parts. Jiao et al. [124,125] established the mathematical model of a CFRTP–stainless-steel laser connection, and carried out the research in the aspects of experiment and application exploration. The wettability and mechanical compatibility of the welding interface were improved by forming a micro-texture on the surface of the light alloy. Liu et al. [126] can efficiently and accurately treat the microstructure of the material surface and improve the bonding strength of al-CFRP by using a laser. Figure 18A shows the micro-nano morphology of CFRP treated by laser. The maximum shear strength of these laser-treated joints is almost four times that of the non-microscopic lapped joints. Figure 19A shows the principle of laser micronano treatment of CFRP (Leone et al. [127]). A pulsed Yb:YAG fiber laser was used to pretreat the CFRP surfaces. It was found that after laser treatment, the contact angle of the surface was significantly reduced, the surface free energy was increased, the surface activity was improved, and the strength of the treated adhesive joint was doubled. Li et al. [128] treated CFRP surfaces using picosecond lasers and excimer ultraviolet lasers. The bonding strength between CFRP and aluminum alloy is improved by increasing the surface roughness and introducing functional groups. The shear strength of materials treated by picosecond infrared laser increased by 346.4%, while that by ultraviolet laser increased by 293.8%. Therefore, picosecond laser has higher processing efficiency and is more suitable for adhesive surface treatment. In addition, the finite element analysis shows that the mechanical interlocking caused by surface roughness increases the bonding strength by only 4%, so chemical bonding plays a leading role in the bonding process.

Second, laser energy is directly used for the connection between composite materials and other materials, and laser cleaning is used to perform surface treatment on the joint of the fiber composite materials before bonding or welding to improve the joint strength [129]. The properties of metal and resin materials differ greatly; direct processing of metal and CFRP does not form an obvious intermediate transition layer. However, stable chemical bonds and compounds are formed under the action of heat; usually, the reactions of metal elements M and CFRP O and C elements to form chemical bonds, MGCO_3_, MgO_1_+x, and other compounds, were produced. Figure 19B shows the connection mechanism between laser and metal [130]. Chemical bonds such as Al–O–C and Ti–O–C are formed at the bonding interface between titanium alloy and CFRP, and complex compounds such as TiO_2_, TiO, and TiC are eventually produced. Figure 18B shows the surface morphology and cutting morphology of a laser-treated CFRP/AA2060 joint [131]. The formation of these compounds plays a very important role in improving the mechanical properties of joints [130,131,132].

Overall, the use of lasers for CFRP material surface treatments such as cleaning, weaving, and other surface treatments, as well as for laser welding and gluing, has emerged as a new area of research. Some nations have achieved significant strides in this area. In terms of laser cleaning, a few countries such as the United States and Germany have realized the laser cleaning application of fiber composite products. Laser paint removal has been used on composite surfaces such as the flat tail of an F16 fighter jet and the propeller blades of US Navy helicopters H-53 and H-56. In terms of process application, Germany adopts the laser connection processing method to process BMW 7 Series models, demonstrating the feasibility of laser connections in new lightweight products.

## 5. Numerical Simulation of CFRP Processing

Due to the limitation of experimental conditions and observation means, it is long and costly to use experimental design to obtain the optimal process parameters for a specific material before processing, and it is limited to studying the complex process of CFRP laser processing only through experiments, so the numerical simulation study of laser processing is an effective means to analyze the material removal mechanism and optimize the process parameters.

Simulation programs like ANSYS, COMSOL, and ABAOUS are frequently used to model the material in 1D, 2D, 3D, and axisymmetric models in the numerical simulation study of laser processing CFRP. Based on the laser and material parameters, as well as the simulation modeling procedure as needed, the following mechanisms are typical: (1) a thermal model, a dual-temperature model for ultrashort pulses and Fourier conduction model for long- and short-pulse lasers; (2) an optical model that establishes a material’s absorption model based on the reflection, transmission, and absorption characteristics of the material; and (3) a thermoelasticity-based thermal stress model.

The first one involves simulating CFRP’s ablation phenomenon, as well as predicting its morphology, processing quality, and degree of ablation while adjusting the laser’s parameters. The assessments of thermal stress distribution, HAZ, and temperature-field change during CFRP laser activity are the second. The two primary categories of material models used in the simulation process are homogeneous material models and heterogeneous material models. The homogeneous material model is to homogenize the carbon fiber and resin matrix in the composite material. The mixing rule [133] and the Eshelby method [134] are two of the more popular ways. To make the calculations easier, both methods make the assumptions that the carbon fibers are evenly distributed and the material is devoid of cavities. A single or several representative volume elements of the heterogeneous material model simulate the carbon fiber/resin microstructure that exists within the material. The Stefan problem, also known as surface ablation with resin matrix pyrolysis, results in two shifting boundaries in the numerical simulation. The raw and dead cell approach, moving mesh method, etc., can be utilized in commercial finite element software to solve the moving boundary problem.

Based on the anisotropic properties of carbon fiber composites, Di et al. [135] constructed a three-dimensional temperature field distribution for laser processing by COMSOL. The impacts of various process parameters on the temperature variation of CFRP and the mechanism of thermal damage production were investigated by Zhou et al. [136]. A physical model for laser processing of CFRP was built based on the experimental results. A “vertical heat flow” model was created by Weber et al. [137] to explain the sublimation process and to calculate the least amount of damage that can be done to the matrix material. A two-dimensional heat flow analysis model was created by Kononenko et al. [138] to anticipate the necessary number of scans to significantly degrade the resin matrix.

Using Open FOAM as a finite volume method (FVM) library, Ohkubo et al.’s [139] 3D numerical simulation of laser processing of carbon fiber composites was completed. The simulation outcomes demonstrate that the processing settings can be changed to maximize the carbon fiber removal from epoxy resin. To create a three-dimensional finite element model for laser isotropic cutting of monofilament organized CFRP, Yu et al. [140] disclosed the mechanism of laser energy absorption and transmission by the material during the laser cutting of carbon fiber composites.

**Figure 20:**
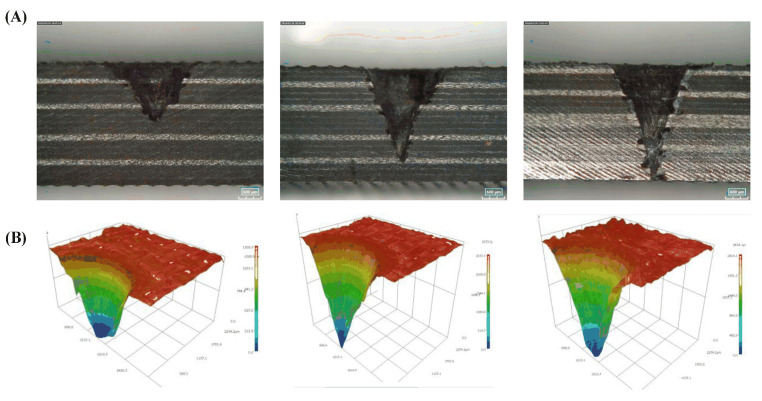
Phenomenon and simulation experiment diagram of processing time of 6, 8, and 10 s in CFRP laser drilling: (**A**) experiment; (**B**) simulation [141].

To create a numerical model of the three-dimensional removal and transient temperature field evolution of the CFRP-simulated material to predict the size of the hole taper, Liu et al. [141] used the finite element method (FEM). They then performed an experimental demonstration, and the results from the simulation and experiment are in good agreement, as shown in Figure 20.

For the first time, Negarestani et al. [142] created a three-dimensional model for the heterogeneous material model that simulates the transient temperature field and subsequent material removal on a non-uniform fiber matrix grid using the finite element (FE) method’s “meta-death” technique. The dimensions of the HAZ during laser processing are likewise predicted by the model.

With a water-jet-guided laser processing technique, Zhang et al. [143] modeled the three-dimensional transient temperature field and material removal of a nonhomogeneous fiber matrix. This was the first time the impact of the laser-pulse duty cycle on the distribution of the material temperature field was examined. A hybrid simulation strategy employing a homogeneous material model for the entire system and a heterogeneous material model for the crucial areas was developed by Li et al. [144].

The current research on the mechanism of CFRP laser processing is not perfect, and there are still errors in predicting the changes of the temperature field by simulation of laser processing, so some scholars have used a special process to measure the changes in temperature, as well as stress, etc., during processing.

To determine the internal temperature distribution of the material during the laser processing of CFRP, Bluemel et al. [145] proposed two methods: first, adding a temperature-measuring paint containing a heat-resistant nickel–chromium alloy to the resin matrix and determining the temperature distribution during processing by observing the distribution of the internal color of the material after processing; second, inserting thermocouples between the layers of the composite material. The second technique involves sandwiching thermocouples between composite layups. By embedding temperature sensors at various distances from the incision and using a heat flow model, P. Mucha et al. [146] calculated the heat transfer loss laser power up to 30%.

A dynamically movable pyrometer measurement point was established by Dittmar et al. [147] that can recognize the signal pattern produced by the carbon-fiber-reinforced surface, i.e., the dynamic pyrometer point enables precise temperature measurements at key locations in complex weld geometries such as bending welds. Rose et al. [148] emphasized the need for monitoring the laser processing process, and process thermography experiments showed that by adopting the detection of the temperature difference between the isotherm of the glass transition and the subsequent exposure cycles, the processing quality could be improved. A video extensometer system and thermocouples were used by Mrzljak et al. [149] to measure strain and temperature to examine the sensitivity of the test procedure and measurement equipment for this use case, as well as the impact on mechanical parameters.

In conclusion, a complete numerical simulation model can help to optimize the laser processing parameters, such as laser energy and scanning speed, to ensure processing accuracy and boost processing efficiency. It can also serve as a suitable reference for the exploration of the processing mechanism. Online measuring and monitoring can be used to better understand the processing environment and raise processing standards.

## 6. Outlook

This article offers a thorough analysis of CFRP laser processing technology, some conclusions, and trends of CFRP laser processing technology are drawn. With its benefits of being quick, non-contact, highly precise, flexible, and efficient, laser processing is currently widely used in the field of CFRP processing. Using laser technology to cut, drill, mill, clean, and weld CFRP is an important means of improving manufacturing efficiency. To further reduce the HAZ and the width of the cut during processing and complete the low-damage, high-precision, high-quality, and industrialized processing of CFRP, the following areas of research can be focused on.

(1) The development of CFRP laser processing technology with great accuracy and efficiency. Short-wavelength lasers with ultrashort pulses enable ultra-precise, high-quality processing of microstructures. With the birth of kilowatt-class high-power picosecond lasers and the further refinement of ultrafast laser mechanisms, in addition to maintaining the existing advantages in processing accuracy and quality, ultrafast laser processing technology, processing efficiency, and the machinable scale (e.g., processing of thicker composite panels) are also expected to be significantly improved. However, when a higher energy flux is used, it still results in a larger HAZ. Therefore, the appropriate laser parameters (repetition frequency, scan path, etc.) also need to be selected, facilitating the cooling of the material during processing, thus improving the quality of processing.
(2) Development of technology for real-time monitoring of parameters that can be changed at any time according to processing results. During laser processing, laser processing parameters and material parameters have a great impact on the quality of processing. During the processing, affected by the material of CFRP (e.g., lay-up angle, material thickness, etc.) and different laser performance (e.g., laser spot diameter, beam quality, repetition frequency, etc.), a change in each parameter results. Both will cause great fluctuations in processing results. Therefore, in response to this situation, a laser processing database can be created, improving and combining artificial intelligence technology.
(3) Development of larger-area and higher-quality laser processing technology. Laser processing usually processes CFRP in the form of a Gaussian pulse with extremely high energy density because of the uneven distribution of Gaussian laser energy and the high laser energy density in some areas. It is prone to severe thermal damage to materials, so the laser in the actual production of processing accuracy cannot be further improved. Compared to Gaussian beams, the flat-top beam form has the advantages of a low pulse overlap rate and uniform light field, so it is more favorable for lasers in CFRP for applications such as micro and nano processing. Therefore, birefringent element shaping, liquid crystal spatial light modulation shaping, and laser intracavity shaping can be explored to shape Gaussian pulses into flat-topped lasers, achieving larger-area and higher-precision machining.
(4) Improving laser processing automation by fusing robotics with laser processing technology. With the development of spacecraft toward large size and super-size, comes the development of lightweight automobiles, aircraft, etc. CFRP is being used more and more in various fields. The increased demand for human resources also leads to an increase in human error; this will lead to a further reduction in the quality of the CFRP secondary process. Therefore, a combination of robotics and laser processing technology can be used, reducing the influence of human operation and improving the processing speed and accuracy of laser processing. At present, traditional laser-welding robots, cutting robots, and other products have appeared on the market; with further research on laser processing, more and more laser processing robots will appear in the processing line.
(5) Development of water-guided processing technology with high cleanliness, high processing quality, and high-cost performance. Due to the high absorption of laser energy by water and the rapid decay of energy, further research is needed to study the decay law of laser in water, simultaneously finding a suitable method of coupling lasers and water jets, reducing the attenuation energy in water and, thus, increasing the efficiency of water-guided laser transmission and further increasing the processing speed. Water-guided laser processing in CFRP can achieve better quality processing because the matrix easily absorbs water and deliquescence. With the further optimization of the hygrothermal properties of the material, the hydro-conductive processing technology still has a good future.
(6) Development of more efficient and thicker processing technology. Concerning laser composite processing CFRP, multi-energy field composite laser material removal technology is gradually emerging. The commonly used energy fields are electromagnetic fields, thermal fields, vibration fields, etc., but the use in industrial applications is costly and requires further research. With the development of 3D printing technology, laser 3D printing of CFRP parts is a new, fast, and cost-effective method of processing that is beginning to emerge, but it still needs further examination.

## Figures and Tables

**Table 1:** Effect of wavelength on the size of the HAZ.

Reference	Year	Wavelength	Pulse Length	Machining Condition	HAZ
[39]	2016	1064 nm 266 nm	6 ns	Average power 39 mW Repetition rate 10 Hz	700 μm 200 μm
[38]	2020	532 nm 355 nm	12 ps	Repetition rate 0~2000 KHZ Maximum average power 90 W	200 μm 70 μm
[52]	2008	10,600 nm 1064 nm 1030 nm	-- Pulse Continuous	Max power 3000 W Average power 300 W Max power 500 W	1200 μm 600 μm 1400 μm
[53]	2012	10,600 nm 1070 nm	8 µs --	Average power 800 W Pulse frequency 20 KHZ Continuous wave power 300 W	1200 μm 650 μm
[56]	2021	266 nm	30 ns	The single pulse energy >1 mJ Repetition rate 1~100 Hz	82 μm

**Table 2:** Effect of pulse width on heat-affected zone.

Reference	Year	Wavelength	Pulse Length	Machining Condition	HAZ
[59]	2020	1064 nm	0.1~1 ms 4~200 ns 10 ps	Max power 300 W Max power 20 W Max power 70 W	600 μm 140 μm 90 μm
[60]	2022	1064 nm	0.1~1 ms 4~200 ns 15 ps	Max power 300 W Max power 20 W Max power 70 W	729.5 μm 44.7 μm 21 μm
[62]	2020	1030 nm	255 fs	Average power 15 W Repetition rate 1–1.1 MHZ	8.52 μm
[63]	2021	1028 nm	290 fs	Max average power 10 W	<10 µm
[64]	2015	--	8 ps	Average power 1.1 KW Repetition rate 300 KHZ	<20 µm
[66]	2019	1064 nm	0.4 ps	Repetition rate 5.0 MHz Flux of pulse 8.0 J/cm^2^	20 µm
[67]	2013	1030 nm	1.5~7.5 ps	Repetition rate 6.3 MHz Flux of pulse 0.75 J/cm^2^	60 μm

**Table 3:** Processing process characteristics.

References	Year	Processes	Processing Efficiency	HAZ	Plate Thickness
[81]	2010	coaxial-trepan drilling technique	middle	50 μm	7 mm
[83]	2021	staggered scanning processing mode	middle	17.9 μm	10 mm
[84]	2021	dual-beam opposite dislocation	middle	60 μm	10 mm
[85]	2022	stepped-parameter parallel-ring	middle	161.7 μm	2 mm
[86]	2013	spiral rotary hole	high	--	5.5 mm
[87]	2021	“double rotation” cutting method	high	60 μm	4 mm

## Data Availability

Not applicable.
